# EphB4/ TNFR2/ERK/MAPK signaling pathway comprises a signaling axis to mediate the positive effect of TNF-α on osteogenic differentiation

**DOI:** 10.1186/s12860-020-00273-2

**Published:** 2020-04-16

**Authors:** Yu Zhang, Chengzhe Yang, Shaohua Ge, Limei Wang, Jin Zhang, Pishan Yang

**Affiliations:** 1 Shandong Provincial Key Laboratory of Oral Tissue Regeneration, Department of Endodontics, School of Stomatology, Shandong University, No. 44-1 Wenhua Road West, Jinan, Shandong Province China; 2 Shandong Provincial Key Laboratory of Oral Tissue Regeneration, Department of Periodontology, School of Stomatology, Shandong University, No. 44-1 Wenhua Road West, Jinan, Shandong Province China; 3 Department of Oral & Maxillofacial Surgery, Qilu Hospital, Institute of Stomatology, Shandong University, No. 107 Wenhua Road West, Jinan, Shandong Province China; 4 Department of Oral Medicine, Qilu Hospital, Institute of Stomatology, Shandong University, No. 107 Wenhua Road West, Jinan, Shandong Province China; 5 Department of Endodontics, School of Stomatology, Shandong University, Jinan, Shandong Province China

**Keywords:** TNF-α, EphB4, Tumor necrosis factor receptor2 (TNFR2), MAPK cascades, Osteogenesis

## Abstract

**Background:**

Low concentrations of tumor necrosis factor-alpha (TNF-α) and its receptor TNFR2 are both reported to promote osteogenic differentiation of osteoblast precursor cells. Moreover, low concentrations of TNF-α up-regulate the expression of EphB4. However, the molecular mechanisms underlying TNF-α-induced osteogenic differentiation and the roles of TNFR2 and EphB4 have not been fully elucidated.

**Results:**

The ALP activity, as well as the mRNA and protein levels of RUNX2, BSP, EphB4 and TNFR2, was significantly elevated in MC3T3-E1 murine osteoblast precursor cells when stimulated with 0.5 ng/ml TNF-α. After TNFR2 was inhibited by gene knockdown with lentivirus-mediated shRNA interference or by a neutralizing antibody against TNFR2, the pro-osteogenic effect of TNF-α was partly reversed, while the up-regulation of EphB4 by TNF-α remained unchanged. With EphB4 forward signaling suppressed by a potent inhibitor of EphB4 auto-phosphorylation, NVP-BHG712, TNF-α-enhanced expressions of TNFR2, BSP and Runx2 were significantly decreased. Further investigation into the signaling pathways revealed that TNF-α significantly increased levels of *p*-JNK, *p*-ERK and *p*-p38. However, only the *p*-ERK level was significantly inhibited in TNFR2-knockdown cells. In addition, the ERK pathway inhibitor, U0126 (10 μM), significantly reversed the positive effect of TNF-α on the protein levels of RUNX2 and BSP.

**Conclusions:**

The EphB4, TNFR2 and ERK/MAPK signaling pathway comprises a signaling axis to mediate the positive effect of TNF-α on osteogenic differentiation.

## Background

As a pleiotropic cytokine, tumor necrosis factor alpha (TNF-α) is primarily produced by activated macrophages and lymphocytes, and can also be detected in endothelial cells and other cell types [1]. The biological activities of TNF-α are mediated through two structurally distinct receptors, tumor necrosis factor receptor 1 (TNFR1) and TNFR2. In osteoblast precursor cells, TNFR1 mediates activation of NF-κB signaling and inhibition of osteogenic differentiation after TNF-α stimulation [2], whereas TNFR2 activation by progranulin (PGRN), a TNFR2 agonist, plays a protective role in osteogenic differentiation and bone regeneration [3, 4]. Furthermore, TNF-α affects behaviors of mesenchymal stem cells (MSCs) and osteoblast precursor cells in a dose and time-dependent manner [2, 5]. Classically, TNF-α is known as an inhibitor of osteoblastic differentiation and an activator of osteoclastogenesis [5–7]. However, opposite findings suggest that TNF-α may be an important inducer of osteogenic differentiation and bone healing [8, 9]. Interestingly during the healing processes of bone fracture, the expression of TNF-α and its receptors, TNFR1 and TNFR2, presents a biphasic pattern. At the early stage after bone injury is created in mouse models, TNF-α expression is mainly found in macrophages and other inflammatory cells, accompanied by the release of secondary signaling molecules to recruit osteoprogenitor cells via chemotactic effect. At the late stage of wound healing, TNF-α expression is mainly detected in osteoblasts and other mesenchymal cells [10, 11], indicating that TNF-α actively participates in bone fracture regeneration [9]. Indeed, according to the literature data and our previous studies, a short-term exposure to low concentrations of TNF-α enhances the osteogenic differentiation of MSCs and osteoblasts, which in turn promotes bone regeneration [12–15] .

Several molecular mechanisms have been reported to play roles in TNF-α-induced osteogenic differentiation [14–16]. For example, TNF-α modulates the expressions and functions of the adenosine A2B receptors (A2BARs), which have been shown to promote osteogenic differentiation of MSCs both in vitro and in vivo [17, 18]. Wnt5a and NF-κB were also reported to be involved in TNF-α-induced osteogenic differentiation [19–21]. Moreover, we found that ephrinB2-EphB4 signaling pathway, which participates in a broad spectrum of biological processes [22–25], at least partly mediates the osteogenic differentiation promoted by low concentrations of TNF-α [16]. However, it is still unclear whether there exists a crosstalk between the TNFR2 and EphB4 signaling pathways to mediate TNF-α-induced pro-osteogenic effect.

In this study, osteoblast precursor cells were stimulated with a low concentration of TNF-α, and expression levels of TNFR2, EphB4 and several osteogenesis-related markers were determined. The potential signaling cascades involved in these biological processes were also investigated. We found, for the first time, that the EphB4/TNFR2 and ERK/MAPK signaling axis partly mediates TNF-α-induced osteogenic differentiation of these pre-osteoblast cells.

## Results

### TNF-α promotes osteogenic differentiation of MC3T3-E1 cells

**Fig. 1:**
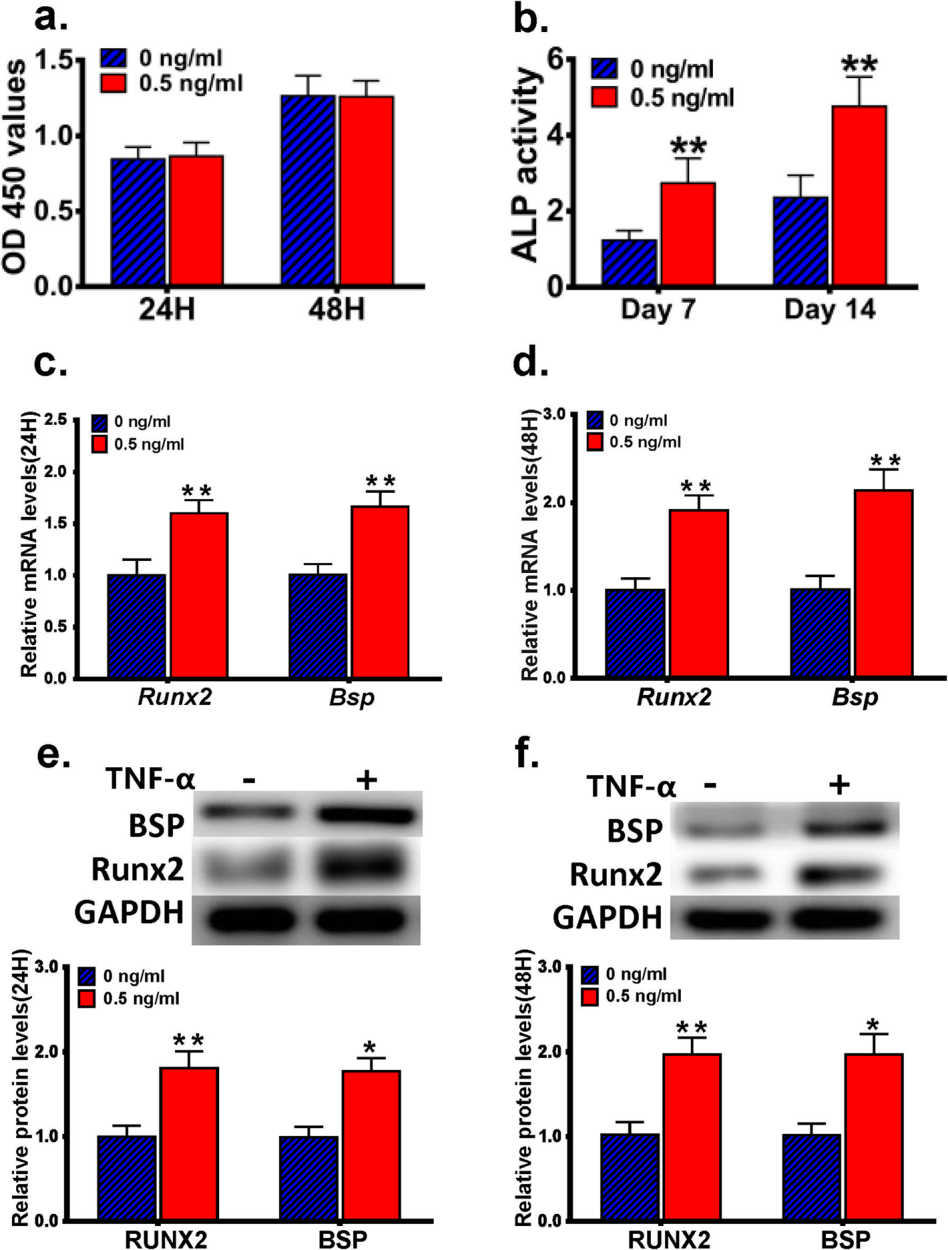
The effect of a low concentration of TNF-α on the proliferation/survival and osteogenic differentiation of MC3T3-E1 cells. **a** MC3T3-E1 cells were treated with or without 0.5 ng/ml TNF-α for 24 h or 48 h, and subjected to CCK-8 assay. No statistically significant difference in cell proliferation/survival was detected in MC3T3-E1 cells treated with 0.5 ng/ml TNF-α when compared with the control cells. **b** MC3T3-E1 cells were cultured in the osteogenic induction medium supplemented with or without 0.5 ng/ml TNF-α for 7d or 14d, and the ALP activity in these cells were determined. **c**, **d** MC3T3-E1 cells were cultured in the osteogenic induction medium supplemented with or without 0.5 ng/ml TNF-α for 24 h (**c**) or 48 h (**d**), and the mRNA levels of *Runx2* and *Bsp* were determined using RT-PCR. **e**, **f** MC3T3-E1 cells were cultured in the osteogenic induction medium supplemented with or without 0.5 ng/ml TNF-α for 24 h (**e**) or 48 h (**f**), and the protein levels of RUNX2 and BSP were determined using western blot analysis. *, *p* < 0.05 vs. the 0 ng/ml group; **, *p* < 0.01 vs. the 0 ng/ml group

MC3T3-E1 cells were treated with or without 0.5 ng/ml TNF-α for 24 h or 48 h, and subjected to CCK-8 assay. No statistically significant difference in cell proliferation/survival was found when compared the TNF-α-stimulated MC3T3-E1 cells with the control cells (Fig. 1a). We then cultured MC3T3-E1 cells in the osteogenic induction medium supplemented with or without 0.5 ng/ml TNF-α for 7d or 14d, and determined the ALP activity in these cells. Our results indicated that TNF-α treatment significantly increased the ALP activity when compared with the control cells (Fig. 1b). We also monitored the mRNA and protein expression levels of RUNX2 and BSP in MC3T3-E1 cells cultured in the osteogenic induction medium supplemented with or without 0.5 ng/ml TNF-α for 24 h or 48 h. We found that the expression levels of RUNX2 and BSP were both significantly higher in TNF-α-treated MC3T3-E1 cells than in the control cells (Fig. 1c-f).

### TNF-α promotes the expression of TNFR2 in MC3T3-E1 cells

**Fig. 2:**
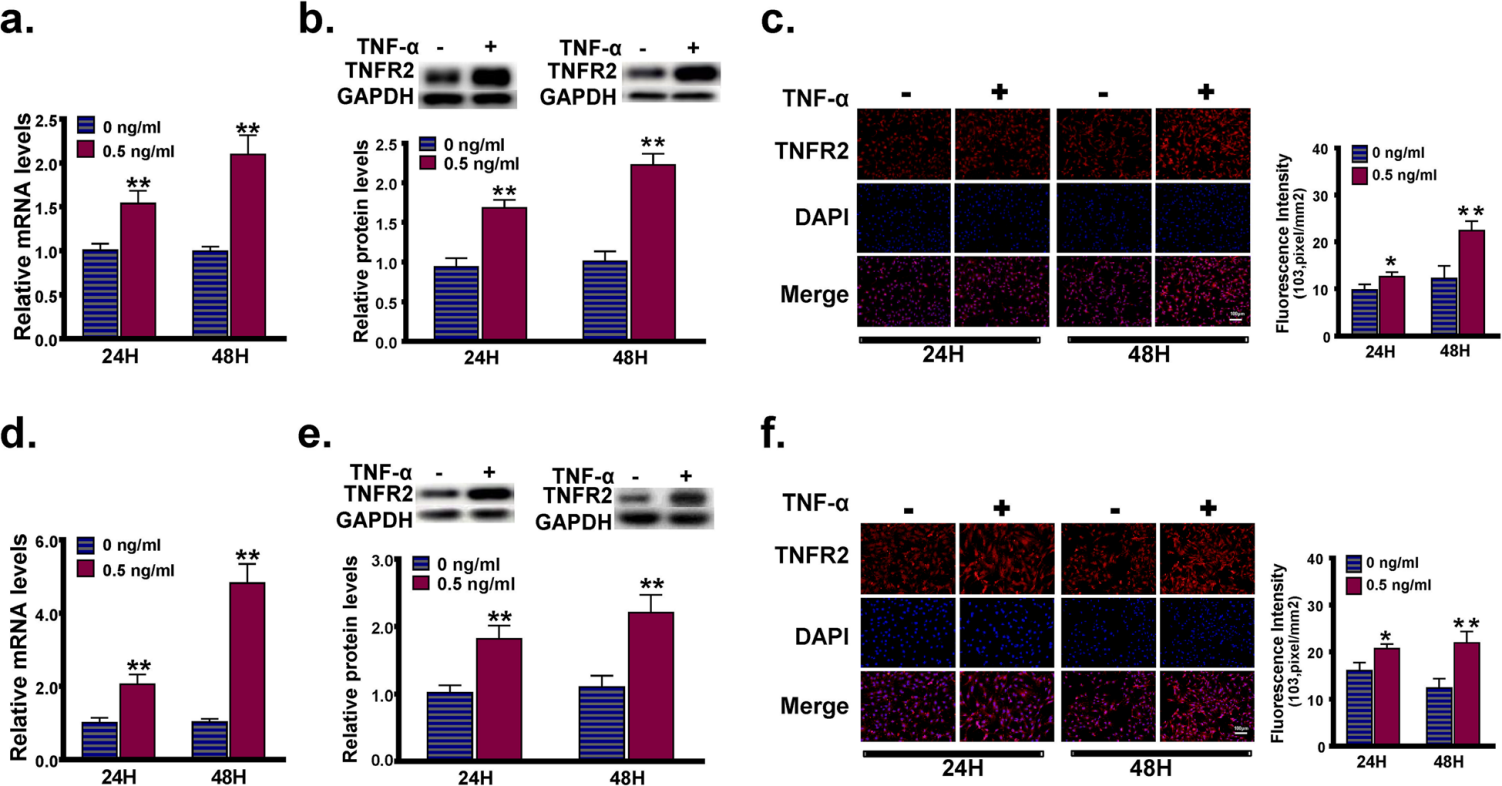
The effect of a low concentration of TNF-α on the expression of TNFR2 in MC3T3-E1 cells. **a-c** MC3T3-E1 cells were treated with or without 0.5 ng/ml TNF-α in the regular culture medium for 24 h or 48 h, and the *Tnfr2* expression level was determined using RT-PCR (**a**), western blot (**b**) and immunofluorescence staining for TNFR2 (**c**). **d-f** MC3T3-E1 cells were cultured in the osteogenic induction medium supplemented with or without 0.5 ng/ml TNF-α for 24 h or 48 h, and the *Tnfr2* expression level was determined using RT-PCR (**d**), western blot (**e**) and immunofluorescence staining for TNFR2 (**f**). *, *p* < 0.05 vs. the 0 ng/ml group; **, *p* < 0.01 vs. the 0 ng/ml groupg

MC3T3-E1 cells were treated with or without 0.5 ng/ml TNF-α in the regular culture medium for 24 h or 48 h, and the expression levels of TNFR2 were determined using RT-PCR, western blot and immunofluorescence staining for TNFR2. Compared with those in the control cells, the mRNA and protein levels of TNFR2 in TNF-α-treated cells were both significantly increased (Fig. 2a, b). As confirmed by the immunofluorescence staining analysis, cells incubated in the presence of TNF-α emitted stronger red fluorescence than the control cells (Fig. 2c). Similar results were obtained in the MC3T3-E1 cells cultured in the osteogenic induction medium supplemented with or without 0.5 ng/ml TNF-α for 24 h or 48 h, indicating that the expression of TNFR2 was also up-regulated by TNF-α stimulation during the processes of osteogenic differentiation (Fig. 2d-f).

### EphB4 mediates TNF-α-promoted expressions of TNFR2 and osteogeneisis-related markers

Our previous study demonstrated that TNF-α significantly enhances EphB4 expression [16]. To investigate the crosstalk between the TNFR2 and EphB4 signaling pathways in mediating the pro-osteogenic effect of TNF-α, we knocked down the expression level of TNFR2 in MC3T3-E1 cells with lentivirus-mediated shRNA interference. Totally 3 siRNAs specifically targeting TNFR2 were designed and synthesized, and the sequences of these siRNAs were listed in Table 1. All of these siRNAs were duplexed, ligated into the pHBLV-Zsgreen-PURO expression vector, and packaged into lentiviral particles. The lentiviral particles were named as pHBLV-TNFR2siRNA1, pHBLV-TNFR2siRNA2 and pHBLV-TNFR2siRNA3, respectively. An siRNA with no homology to any known mouse or human gene was also synthesized, duplexed, ligated into the pHBLV-Zsgreen-PURO expression vector and packaged into lentiviral particles to serve as a negative control. The sequence of the negative control siRNA was also listed in Table 2, and the resulting control lentivral particles were named as pHBLV-NC. MC3T3-E1 cells were then infected with pHBLV-TNFR2siRNA1, pHBLV-TNFR2siRNA2, pHBLV-TNFR2siRNA3 or pHBLV-NC, and the stably transduced cells were selected with puromycin and named as pHBLV-TNFR2siRNA1 cells, pHBLV-TNFR2siRNA2 cells, pHBLV-TNFR2siRNA3 cells and pHBLV-NC cells, respectively.

**Table 1:** Sequences of the synthesized siRNAs and shRNAs specifically targeting murine TNFR2

Control siRNA	TTCTCCGAACGTGTCACGTAA	
**Control shRNA**	**Top**	GATCCGTTCTCCGAACGTGTCACGTAATTCAAGAGATTACGTGACACGTTCGGAGAATTTTTTC
	**Bottom**	AATTGAAAAAATTCTCCGAACGTGTCACGTAATCTCTTGAATTACGTGACACGTTCGGAGAACG
**siRNA1**	GGGACGTTCTCTGACACCACATCAT	
**shRNA1**	**Top**	GATCCGGGACGTTCTCTGACACCACATCATTTCAAGAGAATGATGTGGTGTCAGAGAACGTCCCTTTTTTG
	**Bottom**	AATTCAAAAAAGGGACGTTCTCTGACACCACATCATTCTCTTGAAATGATGTGGTGTCAGAGAACGTCCCG
**siRNA2**	CCACAGTTCTCAGTGCTCTTCCCAA	
**shRNA2**	**Top**	GATCCGCCACAGTTCTCAGTGCTCTTCCCAATTCAAGAGATTGGGAAGAGCACTGAGAACTGTGGTTTTTTG
	**Bottom**	AATTCAAAAAACCACAGTTCTCAGTGCTCTTCCCAATCTCTTGAATTGGGAAGAGCACTGAGAACTGTGGCG
**siRNA3**	CGTGTGAGACTACAGAGACACTGCA	
**shRNA3**	**Top**	GATCCGCGTGTGAGACTACAGAGACACTGCATTCAAGAGATGCAGTGTCTCTGTAGTCTCACACGTTTTTTG
	**Bottom**	AATTCAAAAAACGTGTGAGACTACAGAGACACTGCATCTCTTGAATGCAGTGTCTCTGTAGTCTCACACGCG

**Table 2:** Primer sequences for qRT-PCR

Gene	Forward (5′ ~ 3′)	Reverse (5′ ~ 3′)
***Runx2***	CCCAGCCACCTTTACCTACA	TATGGAGTGCTGCTGGTCTG
***Bsp***	CAGGGAGGCAGTGACTCTTC	AGTGTGGAAAGTGTGGCGTT
***Ephb4***	GCGGAAAGCAACAAAGTA	CGGCAGCGTACAGCATAAGT
***Tnfr2***	CTGCGCCTTGAAAACCCATT	GATGCTACAGATGCGGTGGG
***Gapdh***	AGGTCGGTGTGAACGGATTTG	TGTAGACCATGTAGTTGAGGTCA

**Fig. 3:**
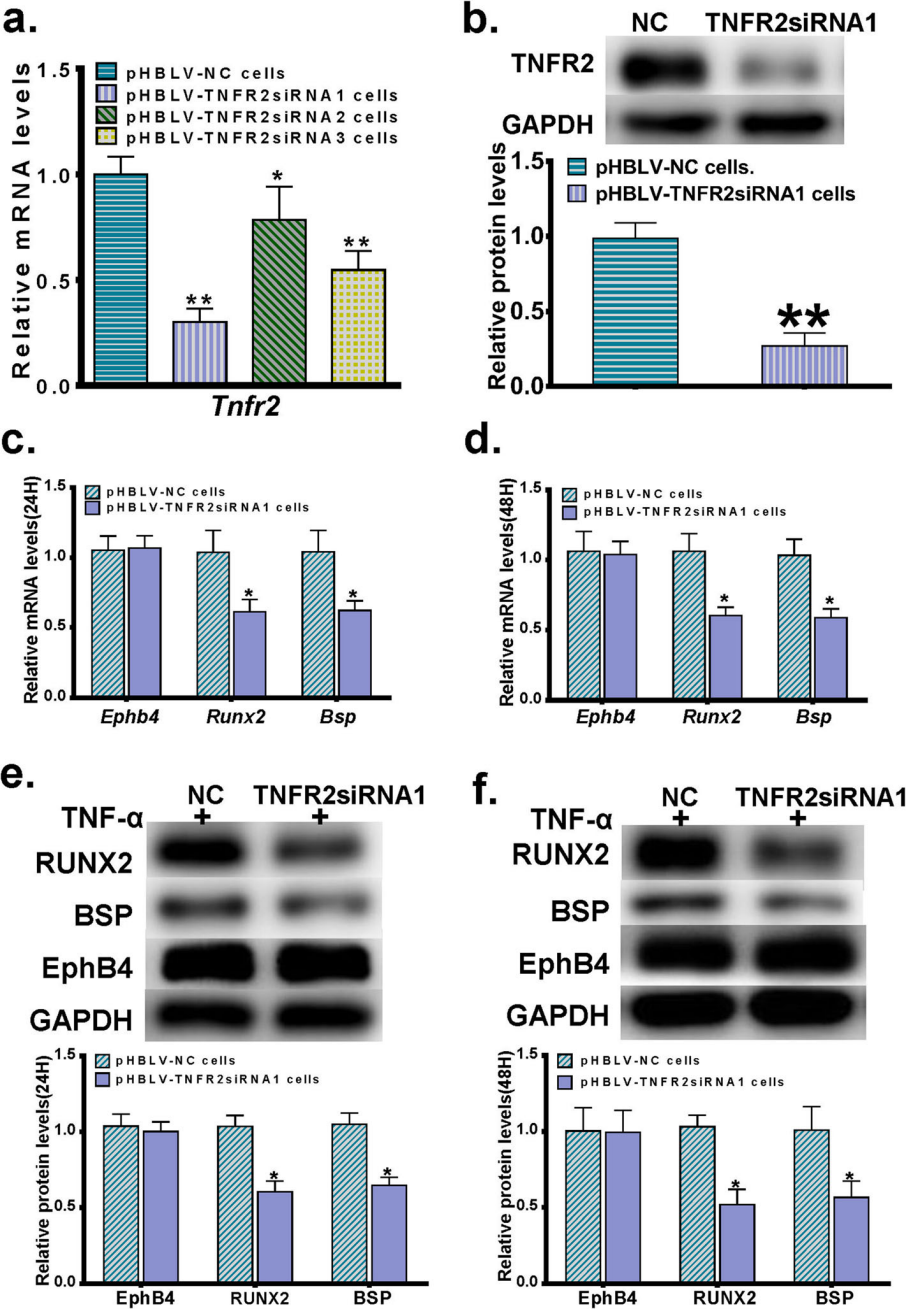
The effect of the lentivirus-mediated shRNA interference of TNFR2 on TNF-α-stimulated EphB4 expression and osteogenic differentiation. **a** MC3T3-E1 cells stably transduced with lentiviral particles were selected with puromycin and named as pHBLV-TNFR2siRNA1 cells, pHBLV-TNFR2siRNA2 cells, pHBLV-TNFR2siRNA3 cells and pHBLV-NC cells, respectively. The mRNA levels of *Tnfr2* were determined in these cells, among which the pHBLV-TNFR2siRNA1 cells displayed the highest TNFR2 gene silencing efficiency and were selected to continue the following studies. **b** TNFR2 protein levels in pHBLV-TNFR2siRNA1 cells and pHBLV-NC cells. **c**, **d** mRNA levels of *Ephb4*, *Runx2* and *Bsp* in pHBLV-TNFR2siRNA1 cells and pHBLV-NC cells cultured in the osteogenic induction medium supplemented with 0.5 ng/ml TNF-α for 24 h (**c**) or 48 h (**d**). **e**, **f** Protein levels of EphB4, RUNX2 and BSP in pHBLV-TNFR2siRNA1 cells and pHBLV-NC cells cultured in the osteogenic induction medium supplemented with 0.5 ng/ml TNF-α for 24 h (**e**) or 48 h (**f**). *, *p* < 0.05 vs. the pHBLV-NC group; **, *p* < 0.01 vs. the pHBLV-NC group

We first detected the mRNA expression levels of TNFR2 in pHBLV-TNFR2siRNA1 cells, pHBLV-TNFR2siRNA2 cells, pHBLV-TNFR2siRNA3 cells and pHBLV-NC cells. We found that the pHBLV-TNFR2siRNA1 cells displayed the highest TNFR2 gene silencing efficiency (Fig. 3a), and thus selected this cell line to continue the following studies. As confirmed by the western blot analysis, pHBLV-TNFR2siRNA1 cells showed an approximately 70% reduction in the TNFR2 protein level when compared with the pHBLV-NC cells (Fig. 3b). To observe the role of TNFR2 in TNF-α-enhanced osteogenic differentiation and EphB4 expression, the pHBLV-TNFR2siRNA1 cells and the pHBLV-NC cells were then cultured in the osteogenic induction medium supplemented with 0.5 ng/ml TNF-α for 24 h or 48 h, and the mRNA and protein levels of EphB4, RUNX2 and BSP were determined. Compared with those in the pHBLV-NC cells, expression levels of RUNX2 and BSP were significantly lower in the pHBLV-TNFR2siRNA1 cells. However, no significant difference was detected in the EphB4 expression levels between the pHBLV-TNFR2siRNA1 cells and the pHBLV-NC cells (Fig. 3c-f), indicating that the TNFR2 activation has no effect on TNF-α-induced EphB4 expression.

**Fig. 4:**
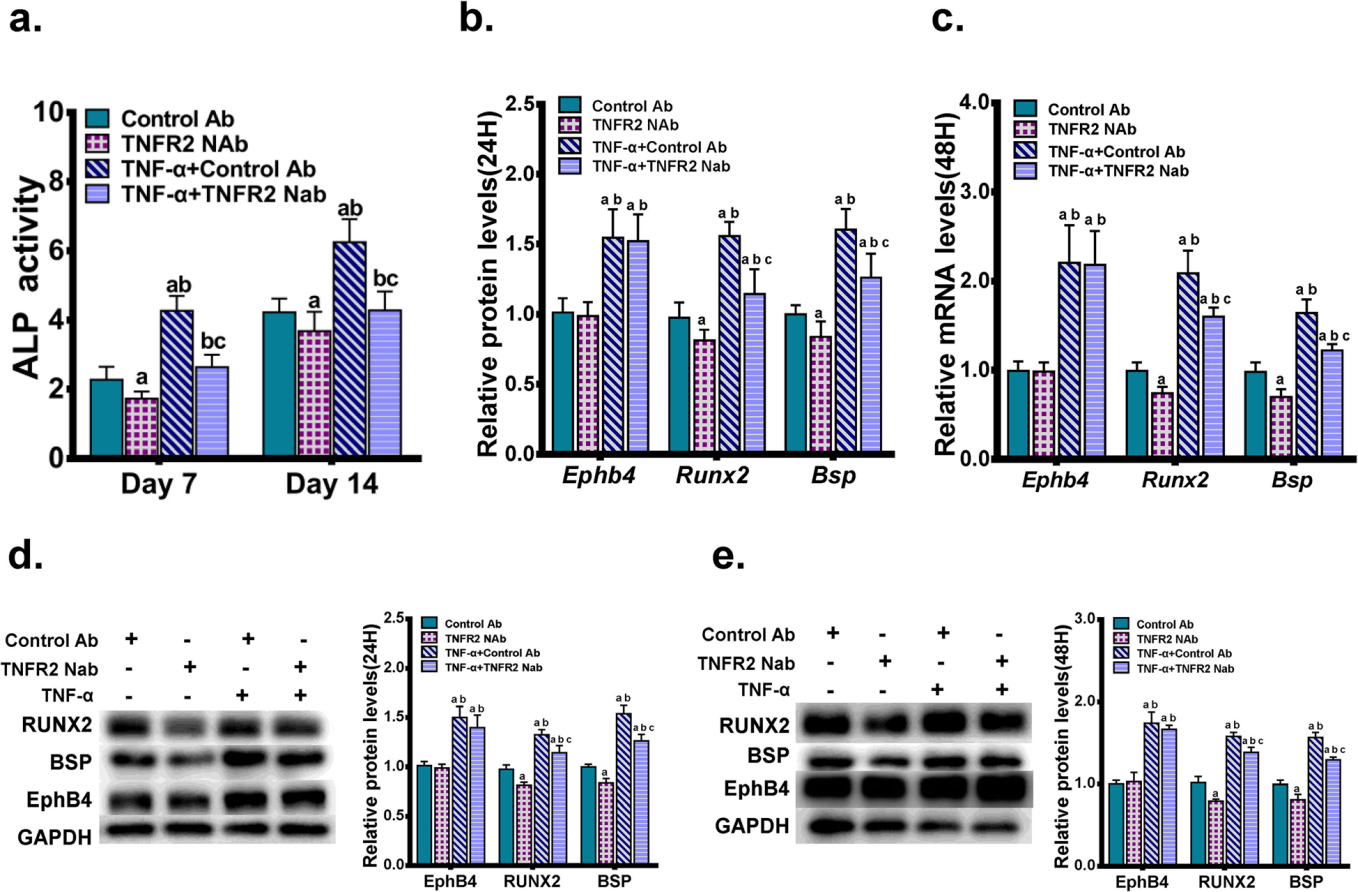
The effect of the impaired binding between TNF-α and TNFR2 on TNF-α-stimulated EphB4 expression and osteogenic differentiation. MC3T3-E1 cells were treated with an anti-mouse TNFR2/ CD120b/TNFRSF1B neutralizing antibody (TNFR2 NAb) at the concentration of 0.2 μg/ml, and were cultured in the osteogenic induction medium supplemented with or without 0.5 ng/ml TNF-α. Cells treated with 0.2 μg/ml of the normal rabbit IgG negative control antibody (control Ab) served as negative controls. (a) ALP activities were determined 7d or 14d after the treatment. (b, c) mRNA levels of *Ephb4*, *Runx2* and *Bsp* were determined after 24 h (b) or 48 h (c). (d, e) Protein levels of EphB4, RUNX2 and BSP were determined after 24 h (d) or 48 h (e). a, *p* < 0.05 vs. the control Ab group; b, *p* < 0.05 vs. the TNFR2 NAb group; c, *p* < 0.05 vs. the TNF-α + control Ab group

To further confirm these results, the binding of TNF-α to TNFR2 was blocked by an anti-mouse TNFR2/ CD120b/TNFRSF1B neutralizing antibody. Briefly, MC3T3-E1 cells were pre-treated with the neutralizing antibody at the concentration of 0.2 μg/ml for 1 h, and were then cultured in the osteogenic induction medium supplemented with or without 0.5 ng/ml TNF-α. Cells treated with 0.2 μg/ml of the normal rabbit IgG negative control antibody served as negative controls. Seven days or 14 days after the treatment, we found that the blockage of the binding between TNF-α and TNFR2 significantly down-regulated the ALP activity enhanced by TNF-α (Fig. 4a). Similarly, 24 h or 48 h after the treatment, the positive effect of the TNF-α treatment on expressions of RUNX2 and BSP was partly reversed by the blockage of the TNF-α-TNFR2 binding. However, the positive effect of TNF-α on the expression of EphB4 showed no changes upon the treatment of the neutralizing antibody against TNFR2 (Fig. 4b-e).

**Fig. 5:**
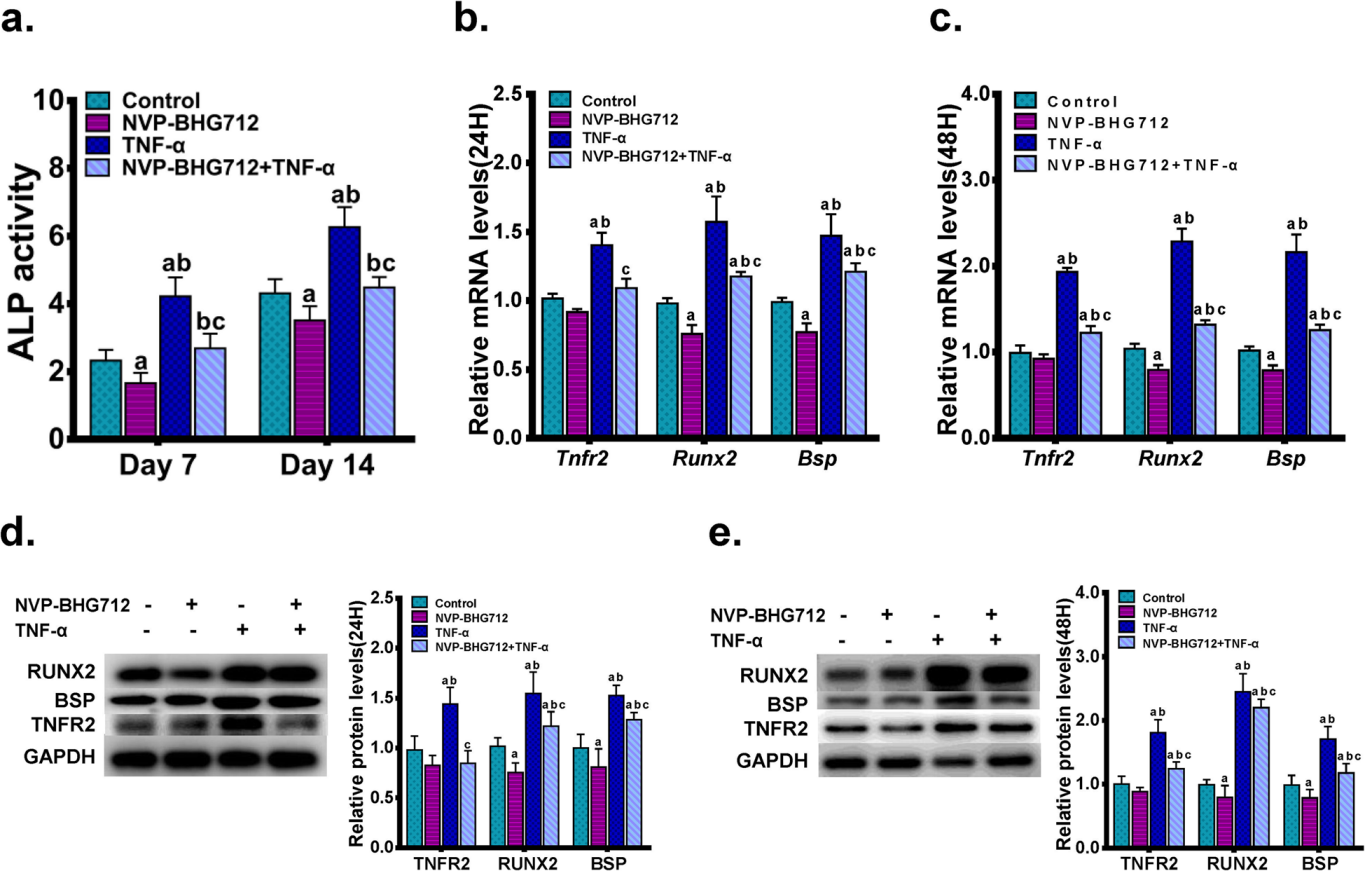
The effect of inhibited EphB4 forward signaling on TNF-α-stimulated TNFR2 expression and osteogenic differentiation. (a) A potent inhibitor of EphB4 auto-phosphorylation, NVP-BHG712, was used to suppress EphB4 forward signaling. MC3T3-E1 cells were pretreated with 200 nM NVP-BHG712 in the regular culture medium for 1 h. Cells were then incubated in osteogenic induction medium supplemented with 200 nM NVP-BHG712 and/or 0.5 ng/ml TNF-α for 7d or 14d. MC3T3-E1 cells cultured in osteogenic induction medium served as controls. The ALP activities were determined. (b, c) MC3T3-E1 cells were pretreated with 200 nM NVP-BHG712 for 1 h in the regular culture medium, and then incubated in osteogenic induction medium supplemented with 200 nM NVP-BHG712 and/or 0.5 ng/ml TNF-α. Cells cultured in osteogenic induction medium served as controls. mRNA levels of *Tnfr2*, *Runx2* and *Bsp* were determined after 24 h (b) or 48 h (c) of incubation. (d, e) MC3T3-E1 cells were pretreated with 200 nM NVP-BHG712 for 1 h in the regular culture medium, and then incubated in osteogenic induction medium supplemented with 200 nM NVP-BHG712 and/or 0.5 ng/ml TNF-α. Cells cultured in osteogenic induction medium served as controls. Protein levels of TNFR2, RUNX2 and BSP were determined after 24 h (d) or 48 h (e) of incubation. a, *p* < 0.05 vs. the control group; b, *p* < 0.05 vs. the NVP-BHG712 group; c, *p* < 0.05 vs. the TNF-α group

To explore the role of EphB4 in TNF-α-enhanced osteogenic differentiation and TNFR2 expression, EphB4 forward signaling was suppressed by a potent inhibitor of EphB4 auto-phosphorylation, NVP-BHG712. Briefly, MC3T3-E1 cells were seeded in 6-well plates at a density of 1.2 × 10^5^ cells per well and were pretreated with 200 nM NVP-BHG712 in the regular culture medium for 1 h. Cells were then incubated in osteogenic induction medium supplemented with 200 nM NVP-BHG712 and/or 0.5 ng/ml TNF-α. MC3T3-E1 cells cultured in osteogenic induction medium served as controls. Seven days or 14 days after the treatment, we found that NVP-BHG712 treatment significantly down-regulated TNF-α-stimulated ALP activity in MC3T3-E1 cells (Fig. 5a). Twenty-four hours or 48 h after the treatment, changes in mRNA and protein expression levels of RUNX2 and BSP demonstrated a similar pattern to that observed in the ALP activity. Interestingly, 0.5 ng/ml TNF-α-stimulated TNFR2 expression was also partly reversed when EphB4 forward signaling was inhibited by NVP-BHG712 treatment (Figs. 5b-e), signifying that TNF-α-enhanced EphB4 signaling up-regulates TNFR2 expression.

### EphB4/TNFR2/ ERK/MAPK signaling pathway comprises a signaling axis to mediate the positive effect of TNF-α on osteogenic differentiation

**Fig. 6:**
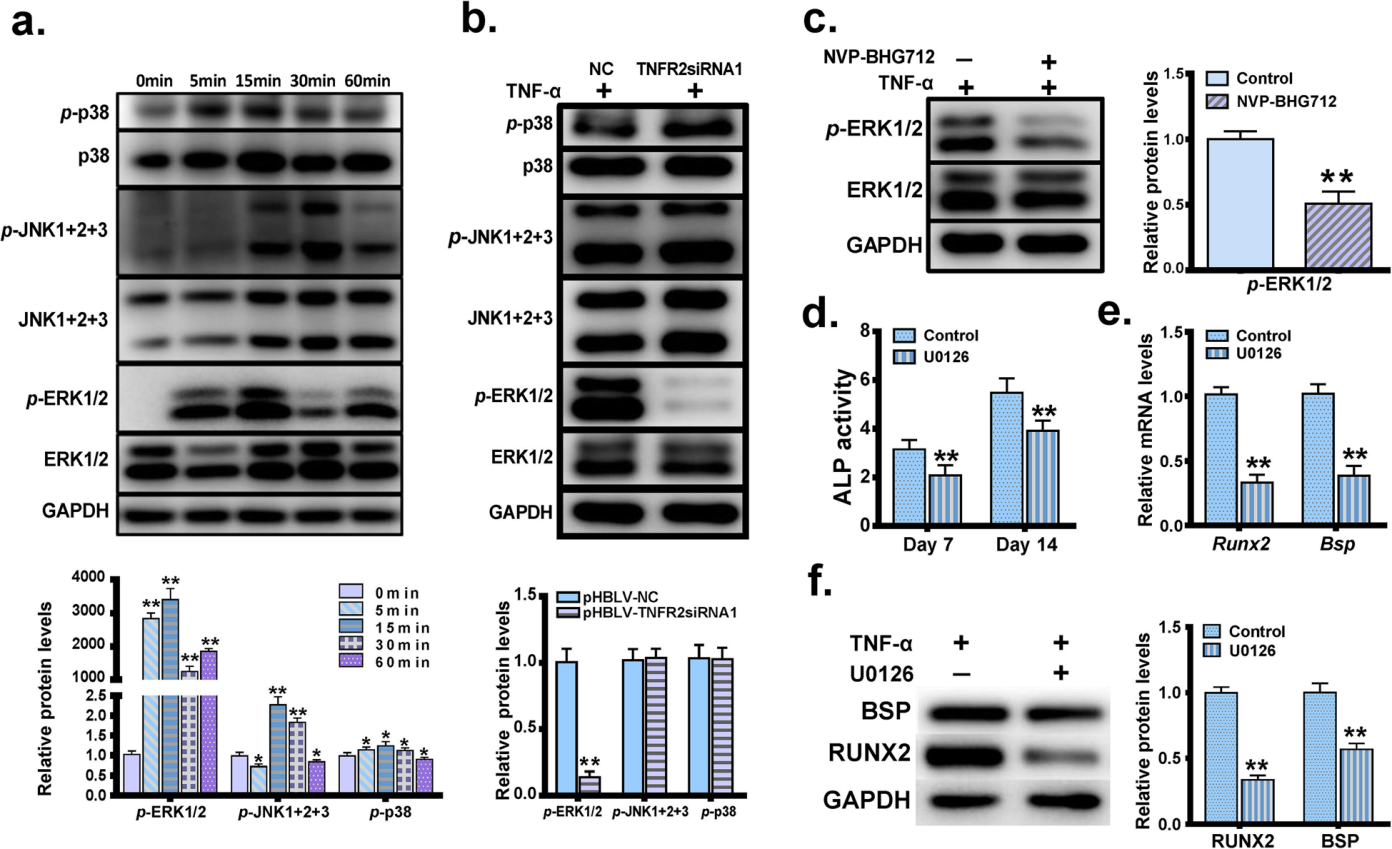
EphB4, TNFR2 and MAPK signaling pathways comprise a signaling axis to mediate the positive effect of TNF-α on osteogenic differentiation. **a** Levels of p38, *p*-p38, ERK1/2, *p*-ERK1/2, JNK1 + 2 + 3 and *p*-JNK1 + 2 + 3 in MC3T3-E1 cells treated with TNF-α for 0 min, 5 min, 15 min, 30 min and 60 min. **b** Levels of p38, *p*-p38, ERK1/2, *p*-ERK1/2, JNK1 + 2 + 3 and *p*-JNK1 + 2 + 3 in the pHBLV-TNFR2siRNA1 cells and the pHBLV-NC cells treated with or without 0.5 ng/ml TNF-α in regular culture medium for 15 min. **c** MC3T3-E1 cells were pretreated with or without 200 nM NVP-BHG712 in the regular culture medium for 1 h, and then 0.5 ng/ml TNF-α was added into the medium. The cells were incubated for another 15 min. Levels of ERK1/2 and *p*-ERK1/2 were determined. **d-f** MC3T3-E1 cells were cultured in the regular culture medium and pretreated with the ERK inhibitor U0126 (10 μM) for 1 h. The culture medium was then switched to the osteogenic induction medium supplemented with 0.5 ng/ml TNF-α and U0126 (10 μM). Cells treated without U0126 (10 μM) served as controls. ALP activities were determined 7d or 14d after the treatment (**d**). mRNA levels (**e**) and protein levels (**f**) of BSP and RUNX2 were determined 3 days after the treatment. *, *p* < 0.05 vs. the control group; **, *p* < 0.01 vs. the control group

It was previously reported that MAPK signaling pathways play an important role in the osteogenic differentiation [26–28]. To investigate whether MAPK members were also involved in TNFR2-mediated pro-osteogenic differentiation upon TNF-α stimulation, MC3T3-E1 cells were first treated with 0.5 ng/ml TNF-α for 0, 5, 15, 30 and 60 min, and levels of p38, *p*-p38, ERK1/2, *p*-ERK1/2, JNK1 + 2 + 3 and *p*-JNK1 + 2 + 3 were monitored using western blot analysis. As shown in Fig. 6a, levels of *p*-p38, *p*-ERK1/2 and *p*-JNK1 + 2 + 3 were all significantly increased after TNF-α stimulation, with the most prominent increase observed at 15 min after TNF-α stimulation. The pHBLV-TNFR2siRNA1 cells and the pHBLV-NC cells were then cultured with 0.5 ng/ml TNF-α in regular culture medium for 15 min, and we found that the *p*-ERK1/2 level was significantly decreased in the pHBLV-TNFR2siRNA1 cells when compared with that in the pHBLV-NC cells (Fig. 6b). After MC3T3-E1 cells were pretreated with or without 200 nM NVP-BHG712 in the regular culture medium for 1 h, 0.5 ng/ml TNF-α was added into the medium and the cells were incubated for another 15 min. Western blot analysis demonstrated that after the EphB4 forward signaling was inhibited, the level of *p*-ERK1/2 was significantly down-regulated (Fig. 6c).

**Fig. 7:**
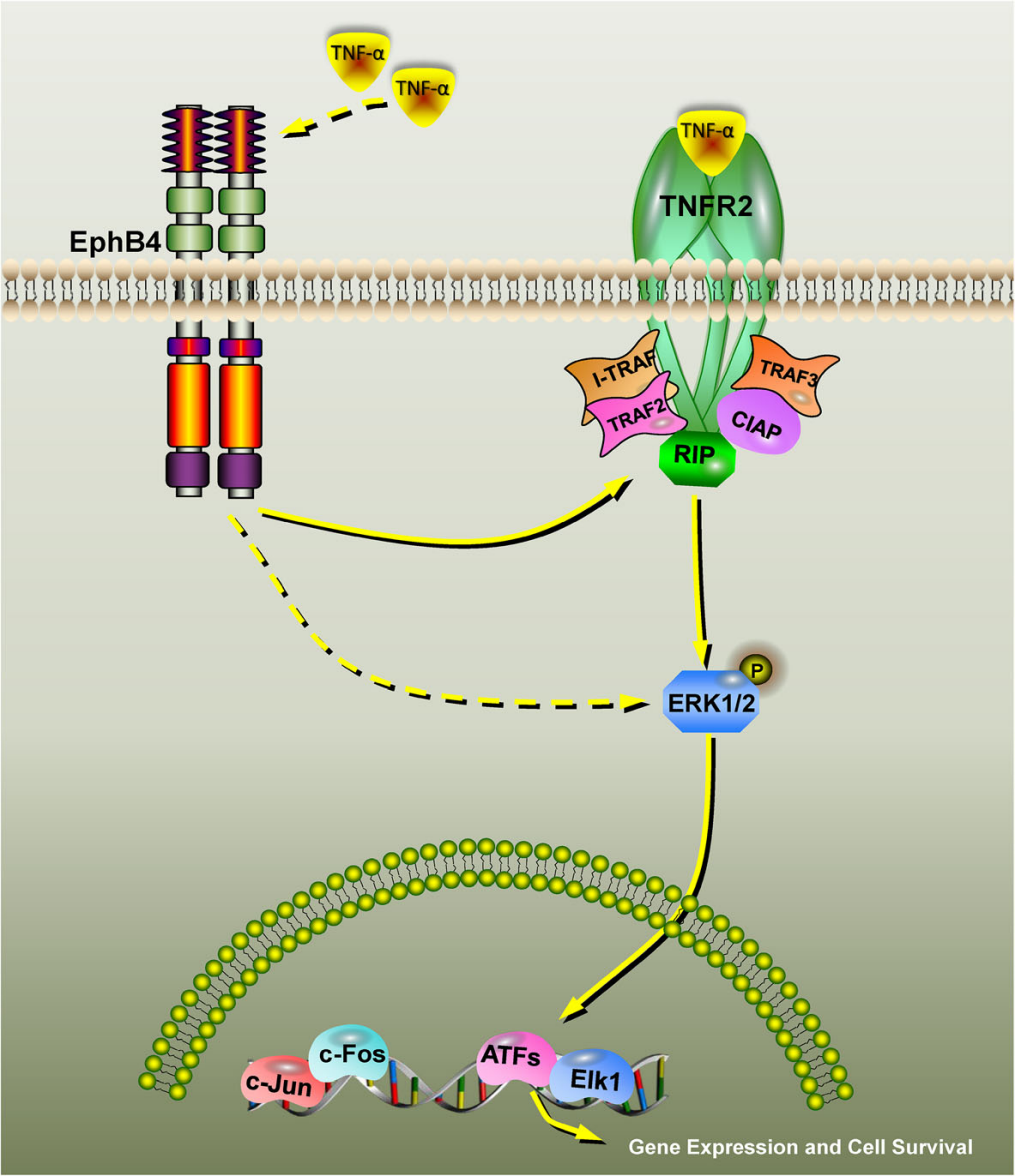
Schematic diagram of the EphB4, TNFR2 and ERK/MAPK signaling pathways. A low concentration of TNF-α first enhances the expression of EphB4, which in turn promoted the expression of TNFR2. The elevated TNFR2 level leads to the activation of the ERK signaling pathway, which eventually enhances the osteogenic differentiation of MC3T3-E1 cells. TNFR2, tumor necrosis factor receptor 2; TNF-α, tumor necrosis factor-alpha; MAPK, mitogen-activated protein kinase; ERK, extracellular signal regulated kinase

To further evaluate the role of ERK signaling in pro-osteogenic differentiation of TNF-α. MC3T3-E1 cells were cultured in the regular culture medium and pretreated with the ERK inhibitor U0126 (10 μM). One hour after the pretreatment, the culture medium was switched to the osteogenic induction medium supplemented with 0.5 ng/ml TNF-α and U0126 (10 μM). Cells treated without U0126 served as controls. Seven days or 14 days after the treatment, we found that U0126 treatment significantly down-regulated the ALP activity enhanced by TNF-α (Fig. 6d). Three days after the treatment, RT-PCR and western blot analysis revealed that the mRNA and protein levels of RUNX2 and BSP were significantly decreased when compared with cells treated with TNF-α only (Fig. 6e, f). These results indicated that EphB4, TNFR2 and the ERK/MAPK signaling pathway comprise a signaling axis to mediate the positive effect of TNF-α on osteogenic differentiation. The schematic diagram of the EphB4, TNFR2 and ERK/MAPK signaling pathways is shown in Fig. 7.

## Discussion

Under physiological conditions, TNF-α exists as a trimer, and binds to either TNFR1 (also known as TNFRSF1A or p55) or TNFR2 (also known as TNFRSF1B or p75) to exert its biological activity. TNFR1 is constitutively expressed on most cell surfaces, whereas TNFR2 gene expression is subject to both transcriptional and post-transcriptional regulation induced by external stimuli [29]. In addition, although the signaling pathways downstream of TNFR1 partially overlap with those of TNFR2, these two receptors often play different and independent roles in a variety of biological activities. For example, TNFR1 mediates cytotoxic effects as well as pro-inflammatory and pro-apoptotic effects, whereas TNFR2 is involved in maintaining the stabilization of Tregs and immunosuppressive facet [30–32]. TNFR1 is also reported to aggravate neuronal tissue destruction in a murine model of retinal ischemia, whereas TNFR2 is found to be effective in promoting neuroprotection [33]. Furthermore, various lines of evidence shows that PGRN-mediated anabolism of chondrocytes and bone formation depend on TNFR2 [3, 34]. Moreover, TNFR2 activation plays a protective role in osteogenic differentiation and bone regeneration [3, 4]. TNFR2 has been reported to play an important role in inflammation control, immune modulation, and neuronal protection. In this study, we investigated the role of TNFR2 in the pro-osteogenic effect of the low concentrations of TNF-α. Consistent with the previous findings showing that TNF treatment preferentially up-regulates TNFR2 on Tregs [35], we found that the TNFR2 expression was significantly enhanced at both mRNA and protein levels in murine pre-osteoblastic cells which were stimulated with TNF-α. Furthermore, lentivirus-mediated shRNA interference of TNFR2 as well as the blockage of the binding between TNF-α and TNFR2 both inhibited the pro-osteogenic effect of TNF-α, strongly indicating that TNFR2 has a pivotal effect on TNF-α-enhanced osteogenic differentiation.

Playing roles in a broad spectrum of biological processes, the ephrinB2-EphB4 bidirectional signaling system has also been reported to substantially contribute to bone remodeling, with the forward signaling through EphB4 into osteoblasts significantly enhancing osteogenic differentiation [22–25]. Our previous study strongly indicated that low concentrations of TNF-α activate EphB4 forward signaling to exert its pro-osteogenic effect in osteoblast precursor cells [16]. Given that both TNFR2 and EphB4 are osteogenic differentiation-associated membrane receptors and their expressions can be promoted by low concentrations of TNF-α, we investigated the interactions between EphB4 and TNFR2 in MC3T3-E1 pre-osteoblastic cells after stimulation with a low concentration of TNF-α in this study. We found that neither lentivirus-mediated shRNA interference of TNFR2 nor the blockage of the binding between TNF-α and TNFR2 showed effects on TNF-α-enhanced EphB4 expression. In contrast, the inhibited EphB4 forward signaling not only inhibited TNF-α-promoted osteogenic differentiation but also lowered TNF-α-stimulated TNFR2 expression, indicating that an activated EphB4 forward signaling is pivotal to TNF-α-enhanced TNFR2 expression and osteogenic differentiation.

In hepatic stellate cells, ephrinB2-EphB4 signaling stimulates ERK-dependent VEGF production and promotes chemotaxis [22]. In the study of ephrinB2-EphB4 signaling between osteoclasts and osteoblasts, ERK was found to be one of the downstream pathways of the EphB4 forward signaling in osteoblasts [23]. In addition, TNF-α activates MAPK signaling pathways including p38, ERK and JNK pathways [36, 37], among which ERK and JNK pathways have been proved to be important pathways for promoting osteogenic differentiation [38–40] . MAPK signaling pathways play important roles in the osteogenic differentiation [26–28]. Consistent with these findings, we found that *p*-p38, *p*-ERK1/2 and *p*-JNK1 + 2 + 3 were all significantly increased in MC3T3-E1 cells stimulated with TNF-α. As indicated by the western blot analysis, disruption of the TNFR2 signaling inhibited the ERK signaling pathway, but showed no inhibitory effect on the p38 and JNK signaling pathways, indicating that the TNFR2 signaling acts upstream of the ERK signaling pathway to promote osteogenic differentiation.

Besides EphB4/TNFR2, other signaling pathways are also involved in TNF-α-induced osteogenic differentiation. For example, TNF-α can modulate the expression and function of A2BAR, which has been shown to significantly enhance osteogenic differentiation [17, 18]. As we have mentioned above, TNF-α can activate p38 signaling, and it has been reported that activated p38/MAPK signaling pathway regulates the expression of BMP-2 in osteoblasts to direct the osteoblastic differentiation of mesenchymal stem cells [14]. Moreover, TNF-α was found to activate the Wnt/β-catenin pathway [41], while the crosstalk between Wnt/β-catenin and the estrogen receptor signaling synergistically promotes osteogenic differentiation of mesenchymal progenitor cells [42]. However, the relationship between the ephB4/TNFR2/ERK/MAPK signaling pathway and those aforementioned signaling pathways in TNF-α-induced osteogenic differentiation needs further investigation.

## Conclusions

Our findings strongly indicate that a low concentration of TNF-α enhances osteogenic differentiation via the activation of a series of signaling events. Briefly, the low concentration of TNF-α firstly stimulates EphB4 expression and enhances EphB4 forward signaling inside the osteoblastic cells, which results in the up-regulation of TNFR2. As a result, the activation of ERK is initiated, which eventually enhances the expressions of bone marker genes and promotes osteogenic differentiation. In summary, EphB4, TNFR2 and the ERK/MAPK signaling pathway comprise a signaling axis to mediate the positive effect of TNF-α on osteogenic differentiation.

## Methods

### Cell culture and reagents

MC3T3-E1 mouse pre-osteoblast cell line, obtained from Shandong Provincial Key Laboratory of Oral Tissue Regeneration, were maintained with 5% CO_2_ at 37 °C in α-MEM (Hyclone, Logan, USA) supplemented with 10% (v/v) fetal bovine serum (FBS; Hyclone, Logan, USA), 100 U/ml penicillin (Solarbo, Beijing, China) and 100 μg/ml streptomycin (Solarbo, Beijing, China). For osteogenic induction, the cells were cultured in α-MEM supplemented with 5% FBS, 100 U/ml penicillin, 100 μg/ml streptomycin, 10^− 8^ mol/l dexamethason (Sigma, St, Louis, MO), 50 mg/l ascorbic acid (Sigma, St, Louis, MO) and 10 mmol/l β-glycerophosphate (Sigma, St, Louis, MO). The medium was switched every two days.

TNF-α was purchased from PeproTech (Rochy Hill, NJ, USA), and the concentration of 0.5 ng/ml was selected based on our preliminary concentration screening study. The neutralizing antibody against TNFR2, anti-mouse TNFR2/CD120b/TNFRSF1B neutralizing antibody (catalog# 50128-RN204), was from Sino Biological (Beijing, China). A normal rabbit IgG, which is an unconjugated rabbit polyclonal antibody and is not directed against any known antigen, was purchased from Cell Signaling Technology (catalog# 2729, Beverly, MA, USA) and used as a negative control. The inhibitor of EphB4 auto-phosphorylation, NVP-BHG712, was obtained from MedChemExpress (HY-13258). The ERK inhibitor U0126 were purchased from Cell Signaling Technology (Danvers, MA, USA).

### Cell proliferation assay

Cell Counting Kit-8 (CCK-8; Dojindo, Kumamoto, Japan) was used to evaluate cell proliferation/survival. Briefly, MC3T3-E1 cells were seeded in 96-well plates at a density of 3 × 10^3^ cells per well and cultured in regular culture medium for 24 h. The medium was then switched to α-MEM supplemented with 0.1% FBS and 0.5 ng/ml murine TNF-α (Peprotech Inc., Rocky Hill, NJ, USA). After 24 h or 48 h of TNF-α stimulation, 10 μl of CCK-8 solution was added to each well and the plates were incubated for 2.5 h at 37 °C. The optical density was measured at 450 nm using the SPECTROstar Nano Microplate Reader (BMG Labtech Inc., Ortenberg, Germany).

### RNA isolation & reverse transcription-polymerase chain reaction (RT-PCR)

Our previous study has proved that low doses of TNF-α promotes expressions of osteogenesis-related markers at 7 and 14 days [16]. Considering that the main aim of this study is to investigate the signaling pathways mediating the pro-osteogenic effect of low concentrations of TNF-α, we selected 24 h or 48 h after osteogenic induction as the observation time points in this study. To determine the mRNA levels, total RNA was extracted from the cells using Trizol® reagent (TaKaRa Biotech, Tokyo, Japan) according to the manufacturer’s instructions, and was reverse transcribed using Reverse Transcriptase (TaKaRa Biotech, Tokyo, Japan). RT-PCR was performed using SYBR® Primix Ex TaqTM (TaKaRa Biotech, Tokyo, Japan) with Roche 480 LightCycler System (Roche Diagnostics, Mannheim, Germany). Each sample was prepared in triplicate, and each experiment was repeated at least 3 times. GAPDH was used as a loading control. The sequences of the primers for amplification of mouse *Tnfr2*, *Runx2*, *Bsp*, *Ephb4* and *Gapdh* were shown in Table 1. The relative gene expression levels were calculated using the 2^-ΔΔCT^ method.

### Western blot analysis

Total cell lysates were extracted from MC3T3-E1 cells by incubation with ice-cold RIPA (Solarbo, Beijing, China) containing 1% PMSF (Solarbo, Beijing, China) for 30 min, and the protein concentrations were measured using a bicinchoninic acid (BCA) protein assay kit (Solarbo, Beijing, China). For immunoblot analysis, 20 μg of protein lysates per sample were denatured in 5 × SDS-PAGE loading buffer (Beyotime, Shanghai, China) at 100 °C for 5 min. The samples were then run on 10% SDS-PAGE gels (Beyotime, Shanghai, China), and transferred to polyvinylidene fluoride (PVDF) membranes (Invitrogen, Carlsbad, CA, UAS) for 1 h at 100 V. The membranes were subsequently blocked with 5% defatted milk for 1 h at room temperature and incubated with the primary antibodies overnight at 4 °C. The anti-mouse primary antibodies used in this study were listed as following: RUNX2 (1:1000, catalog no. 12556S; CST, Danvers, MA, USA), BSP (1:1000, catalog no. 5468S CST, Danvers, MA,USA), EphB4 (1:1000, catalog no. A00690; Boster, China), TNFR2 (1:1000, catalog no. ab19139; abcam, Danvers, MA, USA), p38 (1:1000,catalog no. ab170099; abcam, Danvers, MA, USA), *p*-p38 (1:1000,catalog no. ab195049; abcam, Danvers, MA, USA), JNK1 + 2 + 3 (1:1000,catalog no. ab179461; abcam, Danvers, MA, USA), *p*-JNK1 + 2 + 3 (1:5000,catalog no. ab124956; abcam, Danvers, MA, USA), ERK1/2 (1:10000,catalog no. ab184699; abcam, Danvers, MA, USA), and *p*-ERK1/2 (1:8000,catalog no. ab76299; abcam, Danvers, MA, USA). The membranes were then incubated with an HRP-linked goat anti-rabbit secondary antibody (1:5000, catalog no. 7074P2; CST, Danvers, MA, USA) for 1 h at room temperature. For normalization, defatted milk-blocked membranes were incubated with an HRP-linked anti-mouse GAPDH primary antibody (120,000, catalog no. HRP-60004; Proteintech, Wuhan, China) for 1 h at room temperature. Protein bands were visualized using the Chemiluminescent HRP Substrate (Merck Millipore, Billerica, MA, USA). Quantification of the band intensity was carried out using the Image J Software (NIH, Bethesda, MD, USA).

### ALP activity assay

After osteogenic induction for 7d or 14d, the cell lysates were extracted from the MC3T3-E1 cells using 1% Triton X-100 for 30 min on ice. The cell lysates were centrifuged at 1.2 × 10^4^ g for 5 min at 4 °C, and the ALP activity was evaluated using an Alkaline Phosphatase Assay Kit according to the instructions of the manufacturer (Nanjing Jiancheng Bioengineering Institute, Nanjing, China). ALP activity was calculated according to the concentration of the phenol in a standard well and adjusted according to the protein concentration of each sample.

### Immunofluorescent staining

For immunofluorescent staining, MC3T3-E1 cells were washed with cold PBS, fixed with 4% paraformaldehyde, permeabilized with 0.2% Triton X-100 in 1% bovine serum albumin for 30 min, blocked with 5% bovine serum albumin for 1 h at room temperature, and incubated overnight at 4 °C with polyclonal rabbit anti-TNFR2 antibody (1:100 dilution, 19,272–1-AP, Proteintech, USA). Cells were then incubated with Alexa Fluor 594-conjugated goat anti-rabbit secondary antibody (1:100 dilution, SA00006–4, Proteintech, USA). The cell nuclei were stained with 4, 6-diamino-2-phenylindole (DAPI). Stained MC3T3-E1 cells were visualized under a fluorescent microscope (Olympus, BX51, Japan), and images were captured by a CCD camera (CoolSNAP-Pro cf., Media Cybernetics, USA). The fluorescence intensity was counted in selected merged microscopic images by Image-Pro Plus 6.0 software.

### Lentivirus-mediated shRNA interference targeting TNFR2

siRNAs specifically targeting TNFR2 were designed and synthesized by Hanbio Biotechnology Co., Ltd. (Shanghai, China). An siRNA with no homology to any known mouse or human gene was also synthesized to serve as a negative control. Synthesized siRNAs were duplexed, ligated into the pHBLV-Zsgreen-PURO expression vector (Hanbio Biotechnology Co., Ltd., Shanghai, China), and confirmed by gene sequencing. The resulting lentiviral vectors were then transfected into human 293 T cells with pSPAX2 and pMD2G using Lipofiter™ Liposomal Transfection Reagent (Hanbio Biotechnology Co., Ltd., Shanghai, China). Forty-eight hours and 72 h after the transfection, the supernatant was collected twice, and centrifuged at 2000 g for 10 min to remove cell debris. The lentiviral particles were then concentrated by centrifugation at 82,700푔 for 2 h and resuspended in opti-MEM. After the titration, the lentiviral particles were stored at − 80 °C.

For lentiviral infection, MC3T3-E1 cells were seeded onto six-well plates and cultured in the regular culture medium for 12 h. Cells were then incubated with the corresponding lentiviral particles (MOI = 50) in 1 ml of the regular culture medium supplemented with 5 μg/ml polybrene. Four hours later, another 1 ml of the regular culture medium containing 5 μg/ml polybrene was added to each well. The plates were incubated at 37 °C for 16 h before the medium was switched to the regular culture medium. Forty-eight hours after the lentiviral transduction, green fluorescence (from ZsGreen) was captured by a fluorescence microscope (Olympus IX81, Tokyo, Japan). Stably transduced cells were selected with 8 μg/ml puromycin (Sigma, St, Louis, MO) and were maintained in culture medium containing 2 μg/ml puromycin.

### Statistical analysis

All data were expressed as the means ± standard error of the mean (SEM) from at least three replicates for each experiment. Differences between more than two experimental groups and negative control group were analyzed by one-way ANOVA followed by Tukey HSD comparison test. Variance between two groups was compared by two-way t-test with GraphPad Prism software (version 6, by MacKiev Software, Boston, MA, USA). *p* < 0.05 was considered statistically significant.

## Data Availability

All data generated or analyzed during this study are included in this published article.

## Abbreviations

TNF-α: Tumor necrosis factor alpha
TNFR1: Tumor necrosis factor receptor 1
TNFR2: Tumor necrosis factor receptor 2
PGRN: Progranulin
MSCs: Mesenchymal stem cells
A2BARs: A2B receptors
FBS: Fetal bovine serum
RT-PCR: RNA isolation & reverse transcription-polymerase chain reaction
BCA: Bicinchoninic acid
PVDF: Polyvinylidene fluoride
ALP: Alkaline phosphatase
